# Alterations in bacterial communities, SCFA and biomarkers in an elderly HIV-positive and HIV-negative population in western Mexico

**DOI:** 10.1186/s12879-019-3867-9

**Published:** 2019-03-07

**Authors:** Luz A. González-Hernández, Mariana del Rocio Ruiz-Briseño, Karina Sánchez-Reyes, Monserrat Alvarez-Zavala, Natali Vega-Magaña, Alvaro López-Iñiguez, Julio A. Díaz-Ramos, Pedro Martínez-Ayala, RA Soria-Rodriguez, Moises Ramos-Solano, Jaime F. Andrade-Villanueva

**Affiliations:** 1 HIV and Immunodeficiencies Research Institute, Clinical Medicine Department, CUCS-University of Guadalajara, Guadalajara, Jalisco Mexico; 2 HIV Unit Department, University Hospital “Fray Antonio Alcalde”, University of Guadalajara, Guadalajara, Jalisco Mexico; 3 Molecular Biology in Medicine Ph. D. program, CUCS-University of Guadalajara, Guadalajara, Mexico; 4 Geriatric Department, Antiguo Hospital Civil de Guadalajara “Fray Antonio Alcalde”, Guadalajara, Jalisco Mexico

**Keywords:** HIV-infection, Stool microbiota, Elderly population, Biomarkers, SCFAs

## Abstract

**Background:**

The study of stool microbiota has taken great relevance in the last years, given its role in the maintenance of the intestinal metabolic, physiological, and immunological homeostasis, as well as, its effect over HIV biomarkers levels such as CD4/CD8 ratio, high sensitivity C-Reactive Protein (hs-CRP), related to poor outcomes (rapid progression to AIDS). Several efforts have been made to characterize the gut microbiome. In HIV infection, most of the studies report the presence of a dysbiotic pattern; however, few of them have made an approach in elderly HIV-positive subjects despite the fact that nowadays this subgroup is rising. In this study, we compared the composition of faecal microbiota, Short Chain Fatty Acids (SCFAs), and systemic biomarkers between elderly HIV-positive and HIV-negative subjects.

**Methods:**

A cross-sectional study with 18 HIV-negative controls and 20 HIV-positive patients. The quantification of Bacteroidetes, Firmicutes, Proteobacteria, Actinobacteria, *Lactobacillus*, Enterobacteriaceae, *Bifidobacterium*, *Escherichia coli, Clostridium leptum, Clostridium coccoides* was performed in faecal samples by qPCR. The analysis was performed by calculating the ΔCq of each microorganism using 16S rDNA as a reference gene. Faecal SCFAs were measured by HPLC. The hs-CRP and sCD14 were performed by ELISA.

**Results:**

An increase in the Firmicutes/Bacteroidetes ratio, coupled with a significant increase in the proteobacteria phylum was detected in HIV-positive subjects. In contrast, a decrease in the *Clostridium leptum* group was observed*.* Nevertheless, these elderly HIV-positive patients showed higher levels of total SCFAs mainly by an augmented propionic acid values, compared to HIV-negative subjects. Whereas high levels of hs-CRP were positively correlated with sCD14 in the HIV-positive group.

**Conclusions:**

Alterations in bacterial communities reveals a dysbiotic state related to an unbalance of faecal SCFAs. Therefore, these intestinal conditions might drive an increase of poor prognostic biomarkers in elderly HIV-positive subjects.

## Background

The antiretroviral therapy (ART) has notably enhanced survival among people living with HIV infection. Nowadays, their life expectancy is close that of the HIV-negative population [1]. Nevertheless, persistent inflammation and immune activation, despite sustaining viral suppression with ART, are major challenges of the modern HIV treatment era. Poor prognostic biomarkers typically remain abnormally elevated in many HIV-infected subjects; some of them independently predict all-cause mortality and non-AIDS morbidities including cardiovascular disease as well as immunosenescence (changes to the adaptive immune system that are seen in the very old), elevated levels of high sensitivity C-reactive protein (hs-CRP) and low CD4/CD8 ratio [2, 3].

The American Society of Geriatrics (AGS) and the American Academy of HIV Medicine (AAHIVM) have re-defined the term “elderly” in the HIV-infected population. In the context of people with HIV, all 50 year olds and plus are considered as elders because they present a general attrition of a non HIV infected person ten years older [4]. Several similarities have been found between aging and HIV infection: DNA damage and impairment of repair ability, neuro-endocrine alterations, sarcopenia, and immunosenescence phenomenon (CD8^+^ T cells expansion with incomplete activation (CD28^−^), triggering apoptosis, and their progressive loss) [5].

Increased rates of comorbid illness have been observed in HIV-positive subjects; however, they cannot be completely attributed to the direct effect of the HIV infection itself and probably represent a complex interplay of factors, including differing demographics and socioeconomic status, higher rates of traditional risk factors (tobacco use and alcohol intake), opportunistic infections, co-infections, a chronic inflammation state, and exposure to ART [6].

Main causes of persistent inflammation and immune activation during suppressive ART include residual HIV replication, co-infections with herpes and hepatitis viruses, loss of gut mucosal integrity, and microbial translocation [7].

The major functions attributed to the microbiota are: 1) nutrients absorption and food fermentation, 2) stimulation and maturation of the host immune system, and 3) barrier effects against pathogens [8, 9]. Eighty percent of the identified faecal microbiota in healthy adults can be classified into three dominant phyla: Bacteroidetes, Firmicutes, and Actinobacteria [8]. However, the faecal microbiota is a highly complex and diverse bacterial ecosystem. Within this, there is a hierarchy of (i) dominant bacteria (> 10^9^ Colony Forming Units (CFU)/g)) represented by obligate anaerobes such as: *Bacteroides, Eubacterium, Bifidobacterium, Peptostreptococcus, Ruminococcus, Clostridium* and *Propionibacterium*, *and* (ii) subdominant bacteria (< 10^9^ CFU/g) of the Enterobacteriaceae family, specially *Escherichia coli*, and the genera *Streptococcus*, *Enterococcus, Lactobacillus, Fusobacterium, Desulfovibrio,* and *Methanobrevibacter* [8, 10].

The gut microbiota influences the immune system through their bacterial metabolites like Short-Chain Fatty Acids (SCFAs) [11]. The main SCFAs include, in order of proportion: acetic, propionic, and butyric acid that are produced by fibres fermentation by gut bacteria, particularly by members of the Firmicutes phylum [9]. The SCFAs (specially butyric acid) have an essential role as an energy source for enterocytes in keeping gut barrier integrity, anti-obesogenic effect (decrease adiposity), anti-diabetic effect (improve insulin sensitivity), and preventing colonic cancer [12].

The concentration of SCFAs has been correlated with regulatory T cells numbers, which play a key role in the suppression of inflammatory response [13]; thus, SCFAs have an important role in defining the pro-inflammatory and anti-inflammatory milieu in the intestine [9]. However, a pleiotropic effect has been reported depending of the type of SCFA, receptors activation, cell type, microenvironment as well as the organ and disease [14].

Aging is associated with dysbiosis, which is characterized by a decrease in anti-inflammatory phylum and an increase in pro-inflammatory phylum [15, 16]. There are several studies that describe the microbiome composition in HIV-positive vs HIV-negative subjects with different results, but all of them reflect a dysbiosis in subjects living with HIV, regardless of their age [9].

In general, HIV-infection and aging are associated with lower alpha diversity individually, based in Shannon and Simpson index (diversity and dominance respectively) and higher beta diversity (interindividual taxa differences) when compared to HIV-negative subjects, specifically with a decrease in Firmicutes and an increase in Bacteroidetes phylum [17–19]. It is important to highlight that alpha diversity of microbiome is positively correlated with CD4^+^ T-cell count and inversely correlated with markers of microbial translocation and biomarkers of monocyte activation such as soluble CD14 (sCD14) [19]. Therefore, there is growing evidence of a significant link among host microbiome changes and the aging process, the progression of HIV, and the development of early immunosenescence, secondary to chronic immune activation and a perpetual inflammation [9, 20, 21].

Different types of biomarkers have been associated with several pathologies in elderly population, mainly with cardiovascular disease (CVD) and neurocognitive disorders. Elevated levels of hs-CRP have been related to an increase in mortality (OR = 2.0), independently of classical cardiovascular risk factors [22]. sCD14 is strongly related to impaired neurocognitive test performance in attention and learning domains in HIV-positive subjects [23].

Immune activation combined with a paucity of anti-inflammatory responses generally results in accelerated aging in HIV-infected subjects [9]. Thus, it is important to describe microbiota composition, SCFAs concentration, and systemic biomarkers in elderly HIV-positive subjects (≥ 50 years-old) compared to elderly HIV-negative subjects in Mexican population, which was the objective of this study.

## Methods

### Study population

Cross-sectional (observational study) study including 38 participants who were consecutively recruited: 20 patients from HIV clinic and 18 patients from the Geriatrics Service of a tertiary care university hospital in Guadalajara (a 1000-bed teaching hospital in western Mexico) from November 2016 to March 2017. Subjects were invited to voluntarily participate in the study the same day of their scheduled medical visit, in which the fulfilment of the selection criteria was determined. For the HIV-positive subjects: over 50 years old with more than 2 years of stable ART in virologic control and CD4^+^ count over 200 cell/μL. For the HIV-negative subjects: over 65 years old (this age was set based on the fact that the HIV patients generally present an immunological attrition comparable to the immunological state of a HIV-negative person 10 years older as stated by the AGS and the AAHIVM) and stable body weight. For both groups, it was considered as non-inclusion criteria: the use (30 days before the sample was taken) of antibiotics, probiotics, chemotherapy, or immunotherapy; Body Mass Index (BMI) ≥ 30; diagnostic of Diabetes Mellitus, cancer, or co-infections with HBV or HCV.

Once participants agreed, they underwent a comprehensive geriatric assessment by trained staff utilizing standardized methods. Detailed sociodemographic, immunological and health-related information was also obtained. The Hospital Ethic Committee reviewed and approved the study.

### Covariates

Sociodemographic variables included age, gender, academic level, marital status, and morbidity. All these variables were analysed. BMI was calculated as weight in kilograms divided by the square of height in meters. Individuals were considered as normal from 22.0 to 29.9 kg/m^2^ and overweighed/obese with > 30 kg/m^2^. For comorbidity, participants were asked whether they had a physician’s diagnosis of any of 19 chronic diseases described in the World Health Organization’s International Classification of Diseases (ICD-10) [24, 25].

### Immunological profile

The absolute count of CD4^+^ T cells (cells/μL), count nadir CD4^+^ cells as well as percentage, absolute count CD8^+^ T cells (cells/μL), and the CD4/CD8 ratio were evaluated in HIV-positive subjects with FACSCalibur System Beckton Dickinson BD.

### Viral load evaluation

HIV 1-RNA viral load in plasma was measured through the ROCHE Amplicor HIV-1 Monitor 1.5 Ultrasensitive PCR technique with COBAS Ampli-Prep/Cobas Taqman. Controlled or undetectable HIV infection was considered if the viral load was ≤50 copies/mL under treatment.

### Frailty phenotype

The assessment was based on criteria proposed by Fried and Walston: (i) weight loss, (ii) exhaustion, (iii) grip strength, and (iv) walking speed. Cut-off points for grip strength were defined as below the 25th percentile (11.0 kg for women and 21.0 kg for men), and walking speed was defined as above the 75th percentile (10.5 s). Participants were classified as non-frail when obtaining an overall sum of 0 points; they were considered pre-frail with a score of 1 or 2 points and frail with 3 or more points [26].

### Microorganisms quantification by real time PCR

Thirty-eight faecal samples from elderly patients and controls were collected in sterile vials and were immediately frozen at − 80 °C until their processing. The DNA extraction was performed with QIAamp DNA Stool Mini Kit from QIAGEN according to the manufacturer’s instructions. The microorganism quantification was performed by Real Time PCR in the CFX96 thermocycler by BIO-RAD using optic 96 well plates; the samples were used in a DNA working solution of 10 ng/μL. The primers set concentration was 0.3 μM for Bacteroidetes, Firmicutes, Proteobacterias, Actinobacterias, Enterobacteriaceae, and *Lactobacillus, and* 0.25 μM for 16S rRNA, *Bifidobacterium, Escherichia coli*, *Clostridium leptum* and *Clostridium coccoides*. We used Sso Advance Master Mix by BIO-RAD. The amplification program consisted of an initial 95 °C 3-min denaturalization step, followed by 35 cycles starting with one 95 °C 30 s step, followed by 30 s annealing step, and a final step of 72 °C 30 s. The melt curve was obtained with a gradual 0.5 °C increase starting from 54 °C to 95 °C. Annealing temperatures and sequences for each primer are shown in Table 1. Relative quantification was analysed with the ΔCq of each microorganism, using the 16S rDNA as reference gene.

**Table 1:** Information of the oligonucleotides used for the qPCR analysis

Primer	Sequence (5′-3′)		Temp. (°C)	Prod. size (bp)	Reference
16S rRNA	16S-F	AGTTTGATCCTGGCTCAG	62	500	González et al.; 2012
	16S-R	GWATTACCGCGGCKGCTG			
Bacteroidetes	Bact-F	GGARCATGTGGTTTAATTCGATGAT	63	126	Guo et al.; 2008
	Bact-R	AGCTGACGACAACCATGCAG			
Firmicutes	Firm-F	GGAGYATGTGGTTTAATTCGAAGCA	63	126	Guo et al.; 2008
	Firm-R	AGCTGACGACAACCATGCAC			
Proteobacterias	Prot-F	TCGTCAGCTCGTGTYGTGA	56	170	Bacchetti De Gregoris et al.; 2011
	Prot-R	CGTAAGGGCCATGATG			
Actinobacterias	Acti-F	TACGGCCGCAAGGCTA	57	170	Liang et al.; 2016
	Acti-R	TCRTCCCCACCTTCCTCCG			
Lactobacillus (genero)	Lacto-F	GCGGTGAAATTCCAAACG	56	216	Hermann et al.; 2013
	Lacto-R	GGGACCTTAACTGGTGAT			
Bifidobacterium	Bifi-F	CGGGTGAGTAATGCGTGACC	56	139	Furet et al.; 2009
	Bifi-R	TGATAGGACGCGACCCCA			
*C. leptum*	Clep-F	CCTTCCGTGCCGSAGTTA	60	115	Furet et al.; 2009
	Clep-R	GAATTAAACCACATACTCCACTGCTT			
*C. coccoides*	Ccoc-F	GACGCCGCGTGAAGGA	56	199	Furet et al.; 2009
	Ccoc-R	AGCCCCAGCCTTTCACATC			

### Short chain fatty acids analysis

Faecal SCFAs were determined by high-performance liquid chromatography (HPLC). One gram of frozen stool was weighted and homogenised with vortex in 5 mL of tridistilled water for 3 min; the samples were centrifugated 2300 *g* for 40 min. Subsequently, the supernatants were recovered, and pH was adjusted between 2 and 3 with H_3_PO_4_ 5 M. After this, samples were incubated 10 min at room temperature with constant agitation and filtered with a 0.2 μL of Nylon membrane. Samples were analysed using Acquity Arc at 35 °C, and the flow rate was 1 mL/min; the column Shodex KC-811 was used. Acetic, propionic, and butyric acids were quantified based on the retention times of standards injected and using the linear regression equation with Empower 3 software.

### Hs-CRP and sCD14

Serum samples were immediately stored at − 80 °C. Quantification of hs-CRP and sCD14 was performed using Human high sensitivity C-reactive protein (hs-CRP) and Human soluble cluster of differentiation 14 and sCD14, both by ELISA (CUSABIO®) following the manufacturer instructions.

### Statistical analysis

The data was analysed using Statistical Package for the Social Scientist (SPSS) version 23 and Graph Pad 6 software. Medians, Interquartile Ranges (IQR), mean, and standard deviations were calculated for different variables: age, CD4+ absolute count (cell/μL), RNA HIV-1 viral load (copies/mL), and VACS score. Sociodemographic data was compared with either Student’s t test, Fisher exact, or Chi-square, according to the variable analysed. Correlations between quantitative variables were calculated with Spearman test. The microbial and SCFAs differences between the HIV-positive and HIV-negative groups were estimated using U of Mann-Whitney test. Shapiro-Wilks normality test was significant, so the data distribution was considered non-Gaussian. *p* values ≤0.05 were considered as significant.

## Results

### Study population

The HIV-positive group was composed by 16 men and 4 women, everyone over 50 years old with a mean of 57 ± 5. The CD4^+^ nadir median was 74 (IQR: 39,220) cells/μL. The absolute CD4^+^ median was 409 (IQR: 279,744) cells/μL, the median CD4/CD8 ratio was 0.71 (IQR: 0.37,1.16), a RNA HIV-1 viral load mean of 34 ± 9.4 copies/μL, and a mean of 6 ± 3.5 years of undetectable viral load with a NNRTI regimen (TFV/FTC/EFV, co-formulation). According to the CDC classification system for HIV-infected adults and adolescents, 50 % was classified as category C. HIV-negative group included 11 men and 7 women, all of them over 65 years old with a mean of 68 ± 5 (Table 2).

**Table 2:** Demographic and clinical HIV patients and control group characteristics

	HIV-negative (*n* = 18)	HIV-positive (*n* = 20)	*p* value
Age (years)	68 ± 5	57 ± 5	*p* < 0.05^a^
Gender			
Male	11 (61.1%)	16 (80.0%)	*p* = 0.209^b^
Female	7 (38.9%)	4 (20.0%)	
CD4 T cells count; cell/μL	–	409 (IQR: 279,744)	–
CD4 nadir T cells count; cell/μL	–	74 (IQR: 39,220)	–
CD4/CD8 ratio	–	0.71 (IQR: 0.37,1.16)	–
HIV-1 viral load; copies/μL	–	34 ± 9.4	–
Tobacco Use:			
No risk	3 (16.7%)	5 (25.0%)	*p* = 0.697^b^
Moderate	4 (22.2%)	1 (5.0%)	*p* = 0.170^b^
Moderate/intense	2 (11.1%)	1 (5.0%)	*p* = 0.595^b^
High	1 (5.6%)	1 (5.0%)	*p* = 1.000^b^
No consumption	8 (44.4%)	12 (60.0%)	*p* = 0.332^b^
Alcohol use			
Yes	10 (55.6%)	5 (25.0%)	*p* < 0.05^c^
No	8 (44.4%)	15 (75.0%)	
Drugs antecedent			
Yes	1 (5.6%)	13 (65.0%)	*p* < 0.05^b^
No	17 (94.4%)	7 (35.0%)	
Body Mass Index			
Normal	8 (44.4%)	11 (55.0%)	*p* = 0.513^c^
Overweight	10 (55.6%)	9 (45.0%)	
Waist diameter			
Men			*p* = 1.000^c^
< 90 cm	6 (54.5%)	8 (50.0%)	
≥ 90 cm	5 (45.5%)	8 (50.0%)	
Women			*p* = 1.000^b^
< 80 cm	2 (28.6%)	1 (25.0%)	
≥ 80 cm	5 (71.4%)	3 (75.0%)	
Hypertension			
Yes	1 (5.6%)	2 (10.0%)	*p* = 1.000^b^
No	17 (94.4%)	18 (90.0%)	
Hypercholesterolemia			
Yes	8 (55.6%)	11 (55.0%)	*p* = 0.513^c^
No	10 (44.4%)	8 (40.0%)	
Unknown	–	1 (5.0%)	
Depression PHQ-9			
Yes	1 (5.6%)	1 (5.0%)	*p* = 1.000^b^
No	17 (94.4%)	19 (95.0%)	
IBS QoL	2.8 ± 1.8	2.9 ± 1.8	*p* = 0.860^a^
VACS	15.9 ± 9.3	–	
Fragility score			
No-fragility	9 (50)	5 (25.0)	*p* = 0.179^c^
Pre-fragility	4 (22.2)	14 (70.0)	*p* < 0.05^c^
Fragility	5 (27.8)	1 (5.0)	*p* = 0.467^c^
Albumin; g/dL	4.1 ± 0.4	4.3 ± 0.5	*p* = 0.180^a^
hs-CRP; μg/mL	3.5 ± 2.19	2.12 ± 2.05	*p* < 0.05^a^
sCD14; ng/mL	1131.0 (887.1–1559.3)	819.7 (739.6–1030.2)	*p* < 0.05^d^
SCFAs; mM			
Total	46.17 ± 12.9	57.12 ± 13.49	*p* < 0.05^a^
Acetic acid	3.86 ± 3.79	1.52 ± 1.9	*p* < 0.05^a^
Propionic acid	41.07 ± 15.62	55.05 ± 14.25	*p* < 0.05^a^
Butyric acid	0.99 ± 1.55	0.32 ± 0.41	*p* < 0.05^a^
Firmicutes/Bacteroidetes ratio	1.7 ± 0.62	2.37 ± 0.92	*p* < 0.05^a^

^a^Student’s t-test, ^b^Fisher exact, ^c^Chi square, ^d^U of Mann-Whitney test

### Phylum bacterial stool composition

**Fig. 1:**
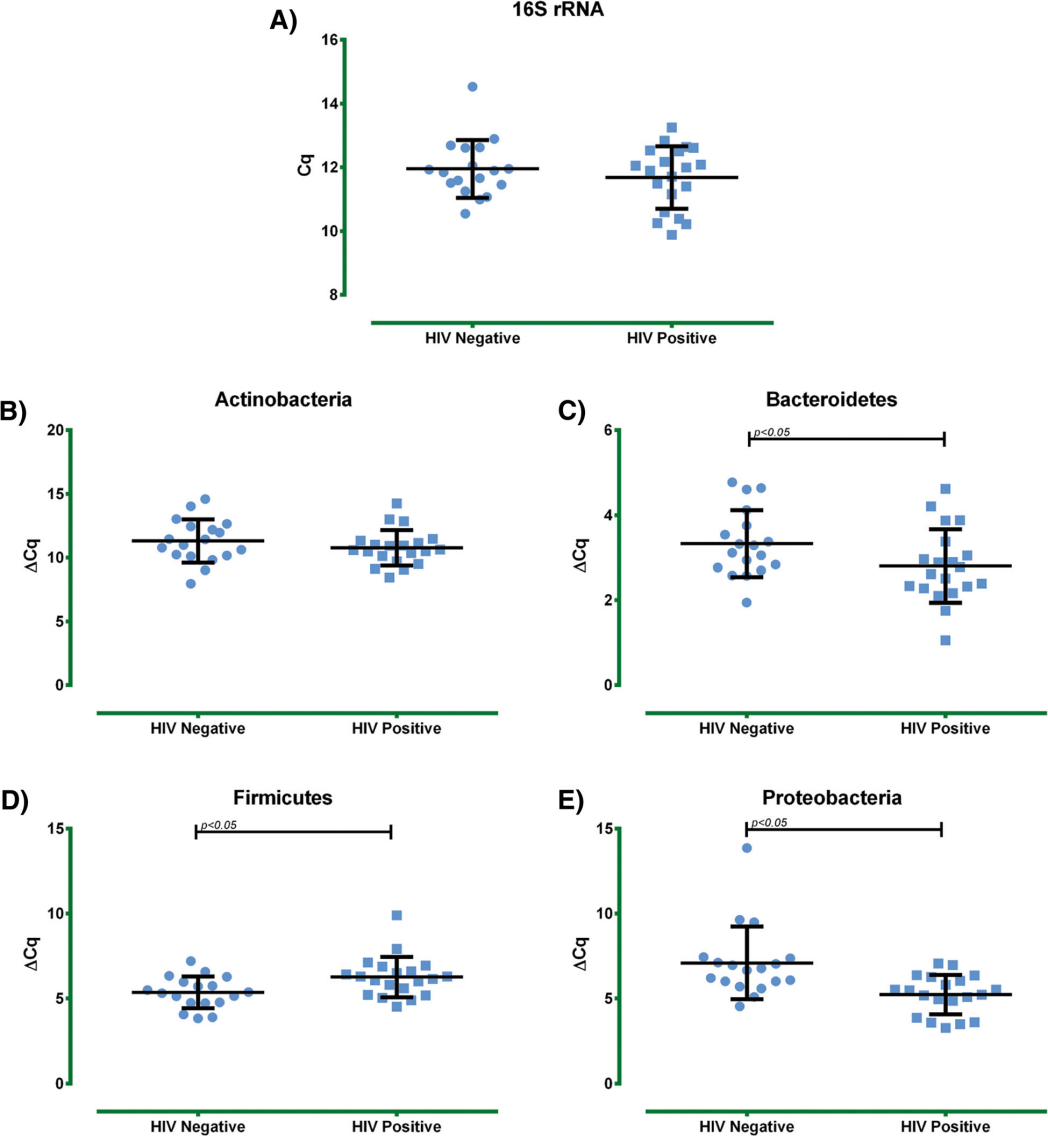
Intestinal bacterial populations assessment by qPCR. Bacterial phylum detections in HIV-positive and HIV-negative elderly subjects were performed by calculation of the delta CT values between tested experimental gene and reference gene. **a**) Universal 16S rRNA raw data. **b**) Actinobacteria, **c**) Bacteroidetes, **d**) Firmicutes, and **e**) Proteobacteria, results were expressed as mean ± SD

The first approach that was taken to know the bacterial composition of the HIV-positive group was to determine different bacterial phyla. As a reference gene, the bacterial 16S rDNA was utilized. No significant difference was found in the universal 16S rDNA quantification between the HIV-positive group and HIV-negative group. Nonetheless, when different bacterial phyla were analysed, we detected a significant decrease in Firmicutes in the HIV-positive subjects compared to the HIV-negative subjects (*p* < 0.05) and a significant increase in the Bacteroidetes phylum (*p* < 0.05) with an evident increase of the Proteobacteria phylum in the HIV-positive group (*p* < 0.001) (Fig. 1). Additionally, a higher Firmicutes/Bacteroidetes ratio was found in HIV-positive subjects compared to the HIV-negative subjects (*p* < 0.05) (Table 2).

### Lactic acid producing genera

**Fig. 2:**
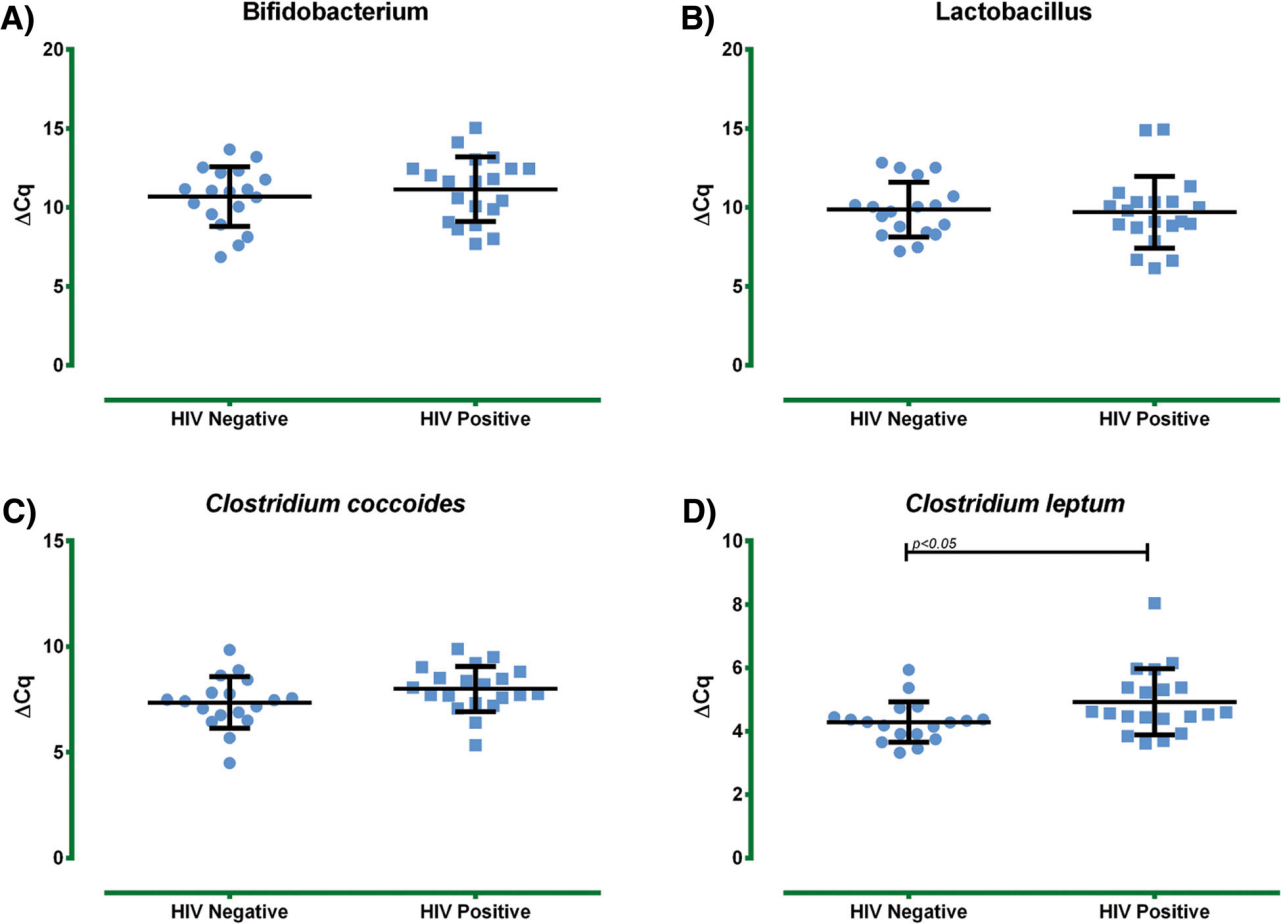
Intestinal commensal bacterial assessment in HIV-positive and HIV-negative elderly subjects. Bacterial determinations were performed by calculation of the delta CT values between tested experimental gene and reference gene **a**) *Bifidobacterium spp*, **b**) *Lactobacillus spp*, **c**) *Clostridium coccoides*, and **d**) *Clostridium leptum,* results were expressed as mean ± SD

Next, the presence of lactic acid producing genera was evaluated: no statistical differences in the amount of *Bifidobacterium* and *Lactobacillus* were detected between both groups (Fig. 2).

### *Clostridium* species: SCFAs producing bacteria

In relation to the amount of SCFAs producing *Clostridium* species, no significant difference was detected for *Clostridium coccoides* (cluster XIVa) between the HIV-positive subjects and the HIV-negative subjects. However, *Clostridium leptum* (cluster IV) showed a significant decrease in the HIV-positive group (*p* < 0.05) (Fig. 2).

### SCFAs levels

**Fig. 3:**
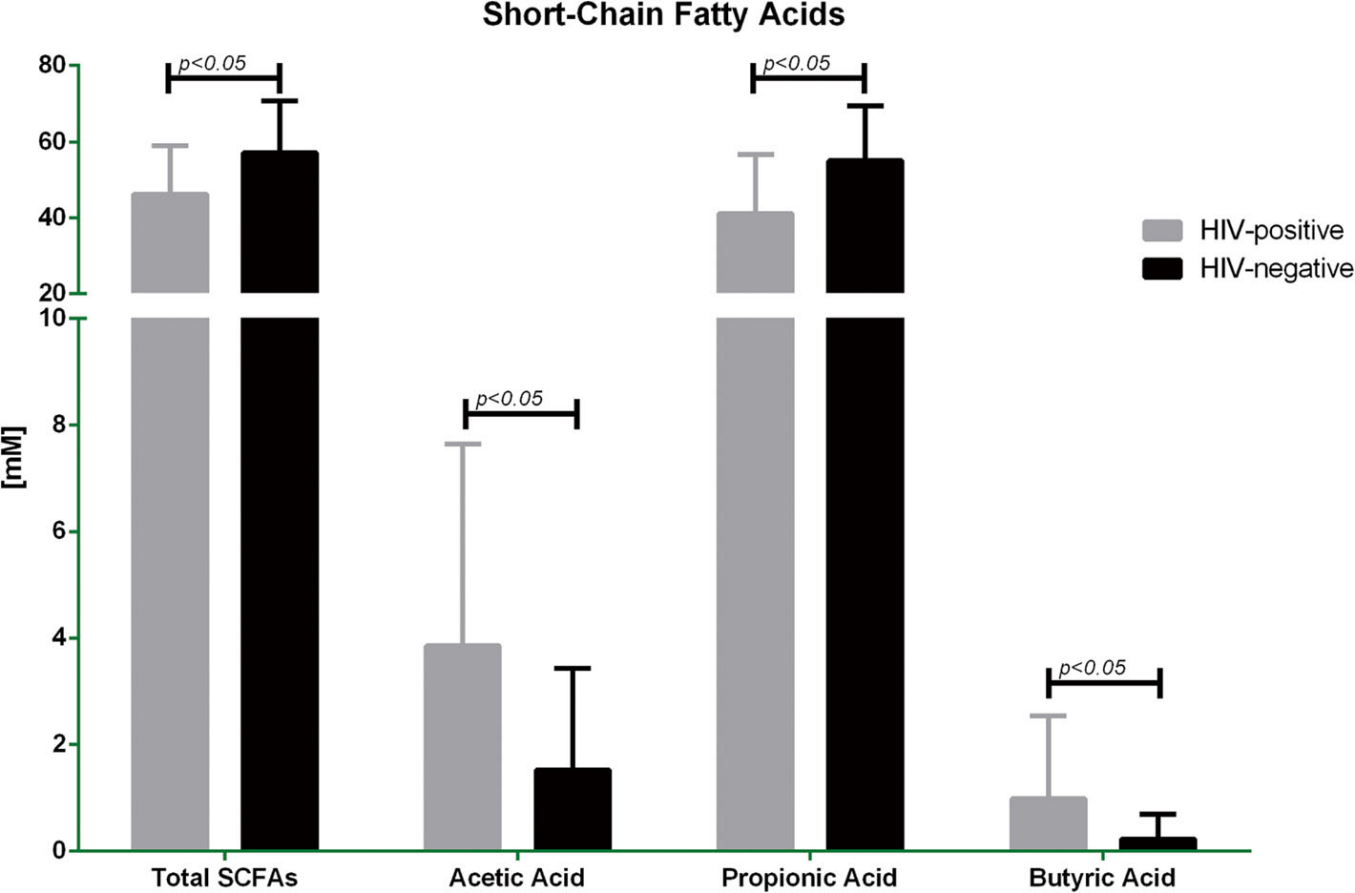
Short Chain Fatty Acids assessment in HIV-positive and HIV-negative elderly subjects. SCFAs levels in faecal samples were determined by HPLC. The figure shows total levels of SCFAs and separate levels of acetic, propionic, and butyric acids. Results were expressed as mean ± SD

Regarding the total quantification of faecal SCFAs, the HIV-positive patients showed higher levels compared to the HIV-negative subjects (*p* < 0.05). A significant increase of propionic acid in the HIV-positive patients versus HIV-negative group was observed (*p* < 0.05), acetic and butyric acid had a significant lower concentration in the HIV-positive group when compared to HIV-negative subjects (*p* < 0.05). Notably, the proportion among SCFAs were altered in both groups (Table 2, Fig. 3).

### Biomarker: Hs-CRP and sCD14 levels

**Fig. 4:**
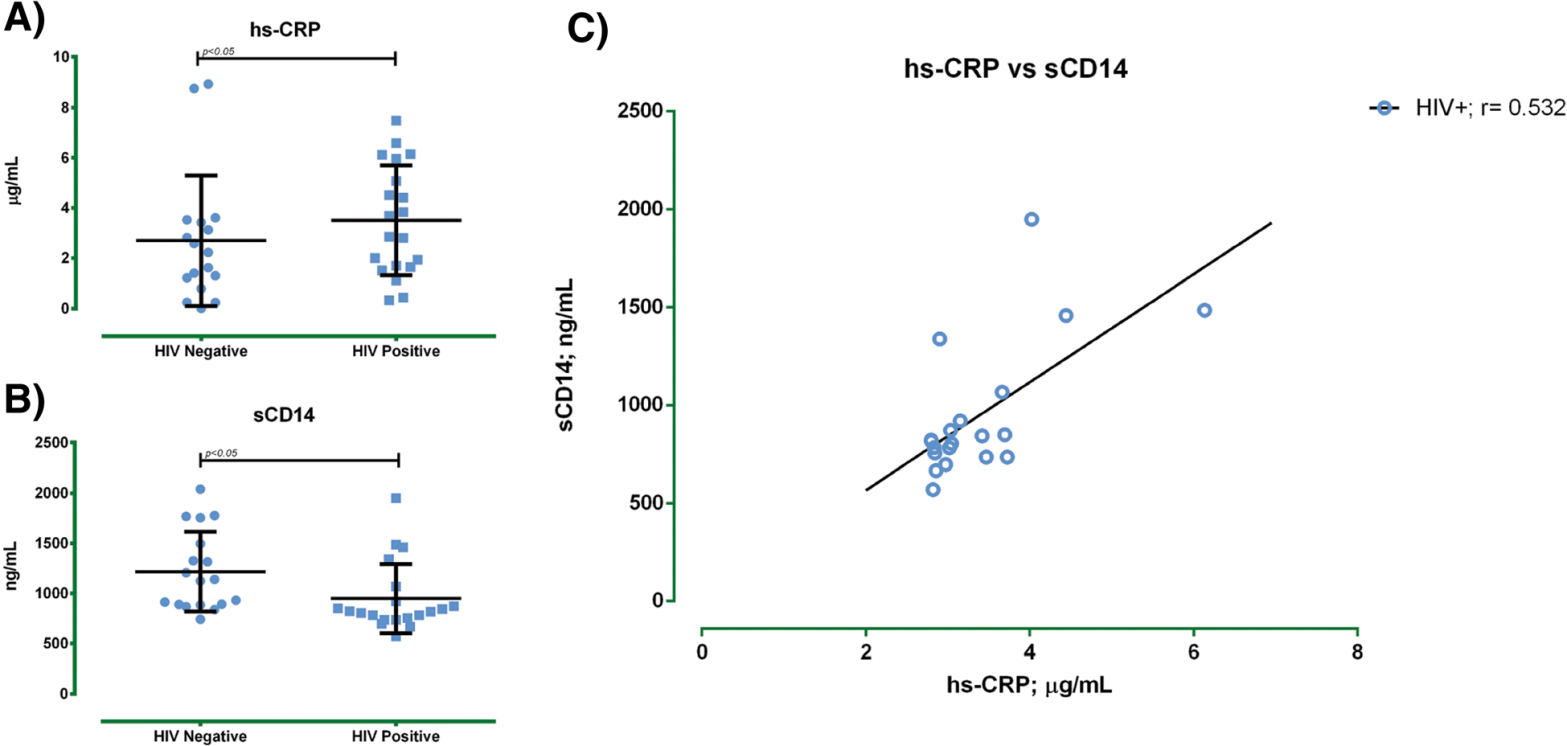
hs-CRP and sCD14 levels in HIV-positive and HIV negative subjects. Serum samples were submitted to sandwich enzyme immunoassay technique (CUSABIO®). **a**) hs-CRP serum levels, **b**) sCD14 serum levels, and **c**) Spearman correlation between hs-CRP and sCD14 in HIV positive subjects Results were expressed as the mean ± SD

As an inflammation biomarker, the media serum levels of hs-CRP were assessed; the HIV-positive group showed a higher level (3.5 ± 2.2 μg/mL) versus the HIV-negative group (2.1 ± 2.0 μg/mL) (*p* ≤ 0.05). Moreover, HIV-positive patients had a lower concentration of sCD14 in comparison with HIV-negative subjects (Table 1; *p* < 0.05). Finally, a positive correlation between hs-CRP and sCD14 was found in HIV-positive group (r = 0.532, *p* < 0.05) (Fig. 4).

## Discussion

Alterations in gut microbiome composition are common during HIV infection; these changes are present at the phyla, genera, and species levels. Previous reports have shown a higher relative abundance in Proteobacteria and Actinobacteria in viremic patients compared to elite controllers [19]. Our data is congruent with these reports; we did observe a significant increase in the Proteobacteria abundance and an increase tendency of Actinobacteria. Therefore, we suggest that the dysbiotic event seems to be perpetuated by the abundance of Proteobacteria.

In untreated HIV patients, a decrease in Bacteroidetes has been reported, which allows the invasiveness of other bacteria such as Proteobacteria [27]. Differently, ART use promotes the increase of the Bacteroidetes phylum and, as a consequence, a decrease of Proteobacteria [19]. This behaviour is opposite to our results considering that we found a significant increase of Bacteroidetes and Proteobacteria. This might be related to duration and kind of ART, immune reconstitution, as well as metabolic and physiological challenge established not only by the HIV infection but also by age; therefore, the high proportion of Proteobacteria, even in the presence of ART, might represent a new behaviour in the microbiota dysbiosis in elderly HIV-positive subjects.

Furthermore, one of our results showed a shift from Firmicutes to Bacteroidetes that is clear and consistent with the previous reported data for elderly individuals [28]. The depletion in the Firmicutes phylum is evident in subjects infected with HIV before ART [29]; others have reported a maintained change in the Firmicutes/ Bacteroidetes ratio in HIV-positive patients even after effective ART [30]. Our data shows that the ratio Firmicutes/Bacteroidetes is significantly higher in elderly HIV-positive patients compared to controls.

At the genus level, we observed a heterogeneous *Lactobacillus* load pattern and a tendency to decrease in Bifidobacterium in HIV-positive subjects. Gonzalez-Hernandez et al.*,* reported that lower counts of *Lactobacillus* are found in faeces of HIV-infected subjects [31]. Several studies have linked the presence of this genus to higher counts of CD4^+^ T cells in HIV-infected individuals [32]. Moreover, it has been reported that *Bifidobacterium* and *Lactobacillus* are depleted from early HIV infection resulting in a deteriorate gut barrier and an inefficient immune function in the GALT [33]. This data promotes the use of *Lactobacillus* as important probiotics given their anti-inflammatory commensal behaviour. The increase in this genus is also related to lower microbial translocation and a better immunological state [34–36]. Thus, we compared the abundance of *Lactobacillus* and CD4^+^ T cells counts and did not observe a correlation (data not shown). This may be due to the fact that other studies were performed in a wide age range of subjects [37], and this study included only elderly subjects.

*Lactobacillus* and *Clostridium* genera are part of the Firmicutes phylum, and in our study a decrease in the *Clostridium* was detected. Since we observed a decrease in Firmicutes, but not obvious changes in the *Lactobacillus*, it can be considered that this dysbiosis is caused by the evident decrease in *Clostridium leptum* and the tendency to diminish in *Clostridium coccoides*. Regarding the age and in accordance with our results, it was reported that in Japanese and Italian population the counts of *Clostridium coccoides* in elderly population were lower compared to adults or young children [38, 39].

The reduction of *Clostridium* implies a destabilization in gastrointestinal health due to the fact that commensal *Clostridium* is strongly involved in the maintenance of overall gut function [40]. Determination of *Clostridium* clusters IV and XIVa has not been reported in elderly HIV-positive population, while our study shows an important decrease in both clusters. Notably, this is the first study that measures the abundance of *Clostridium coccoides* and *Clostridium leptum* in elderly HIV-positive patients.

Additionally, we found a significant increase in total SCFA and an altered proportion among propionic, acetic, and butyric acids compared to healthy gut [41]. Interestingly, this is the first study that shows the proportions of the main SCFA in HIV-positive patients. Diversely, several studies point out that total faecal propionate concentration is related to relative abundance of Bacteroidetes and Proteobacteria [42, 43].

SCFAs have an important role in maintenance of intestinal and immune homeostasis, predominantly related to an anti-inflammatory effect (butyric>propionic>acetic acids) [44]. We found variations in SCFAs proportions, and it has been previously reported that this could be caused by the increase of Proteobacteria and Firmicutes/Bacteroidetes ratio [45, 46]. Therefore, in elderly HIV-positive subjects, the alteration in SCFAs concentrations could contribute considerably to intestinal damage and systemic inflammation.

Interestingly, in our study, 50 % of HIV-positive subjects exhibited very high levels of hs-CRP (> 3 ng/mL) compared to the 22 % in control group, with a positive correlation with sCD14 (*r* = 0.532). Furthermore, 35 % of HIV-positive subjects presented a ≤ 0.4 CD4/CD8 ratio. Both conditions are related to all-cause mortality and comorbidities. In our study, we found a higher incidence in alcohol consumption between HIV-positive and the control group 25 vs 55.6% respectively (*p* < 0.05). Interestingly this data correlated positively with higher levels of sCD14. Suthat Liangpunsakul, et al. in 2017 describe how there is a positive correlation between alcohol consumption and the increase in sCD14 levels. This is even more observed when combined with alcohol consumption there is a high intake of red and processed meats, high-fat dairy products (e.g., whole milk and cream), refined grains, and desserts [47]. Zuleika Michelini, et al. in 2016, presented a similar result. In this study sCD14 levels were compared between HIV-positive and HIV-negatives subjects with Ulcerative Colitis. The sCD14 levels found between these two groups were similar to the levels found in our study [48].

In this sense, it is important to consider that a hallmark in the progression of disease in HIV patients is the chronic immune activation, localized and systemic. This could be secondary to the enteropathy caused by HIV infection or by the use of ART as well as by the bacterial translocation from the intestinal lumen to the circulation. In consequence, elderly HIV-positive patients may have constant immune stimuli that contribute to the maintenance of a low-grade chronic immune activation, biomarkers increase, and promotion of a faster assessment of non-AIDS related comorbidities.

A limitation to this study was the number of patients recruited, we consider that a multi-centre study with the same design as this one, and a higher number of elderly HIV- positive subjects, could provide stronger evidence of the dysbiotic changes that we found in our study. It would be of great interest to further assess these changes on a more complex study design, which includes different age groups.

## Conclusions

This report provides a new understanding of the changes in the gut microbiota and enriches the knowledge of dysbiotic features in the elderly HIV-positive subjects in Mexico. Additionally, it demonstrates the importance to study specific changes in our population and exposes a necessity of deeper and longer studies along with different HIV age groups. The afore-mentioned highlights the need for the development of panels for the detection of the dysbiotic process and the development of clinical studies that assess intervention strategies that allow the re-establishment of intestinal microbiota homeostasis and a reduction of chronic inflammation markers.

## Abbreviations

AAHIVM: American Academy of HIV
AGS: American Society of Geriatrics
ART: Antiretroviral therapy
BMI: Body Mass Index
CFU: Colony Forming Units
EFV: Efavirenz
FTC: Emtricitabine
HIV: Human Immunodeficiency Virus
hs-CRP: High Sensitivity C-reactive Protein
IBD: Inflammatory Bowel Disease
NNRTI: Non Nucleoside Reverse Transcriptase Inhibitor
sCD14: Soluble Cluster of Differentiation 14
SCFAs: Short-Chain Fatty Acids
TFV: Tenofovir
T-reg: Regulatory T cells
VACS index: Veterans Aging Cohort Study index
