# Correction to: Alterations in bacterial communities, SCFA and biomarkers in an elderly HIV-positive and HIV-negative population in western Mexico

**DOI:** 10.1186/s12879-020-05508-3

**Published:** 2020-10-16

**Authors:** Luz A. González-Hernández, Mariana del Rocio Ruiz-Briseño, Karina Sánchez-Reyes, Monserrat Alvarez-Zavala, Natali Vega-Magaña, Alvaro López-Iñiguez, Julio A. Díaz-Ramos, Pedro Martínez-Ayala, R. A. Soria-Rodriguez, Moises Ramos-Solano, Jaime F. Andrade-Villanueva

**Affiliations:** 1 HIV and Immunodeficiencies Research Institute, Clinical Medicine Department, CUCS-University of Guadalajara, Guadalajara, Jalisco Mexico; 2 HIV Unit Department, University Hospital “Fray Antonio Alcalde”, University of Guadalajara, Guadalajara, Jalisco Mexico; 3 Molecular Biology in Medicine Ph. D. program, CUCS-University of Guadalajara, Guadalajara, Mexico; 4 Geriatric Department, Antiguo Hospital Civil de Guadalajara “Fray Antonio Alcalde”, Guadalajara, Jalisco Mexico

**Correction to: BMC Infect Dis 19, 234 (2019)**

**https://doi.org/10.1186/s12879-019-3867-9**

Following publication of the original article [1], the authors identified an error in the labelling of Fig. 3.

in Fig. 3. The correct figure is given below.

**Figure 1:**
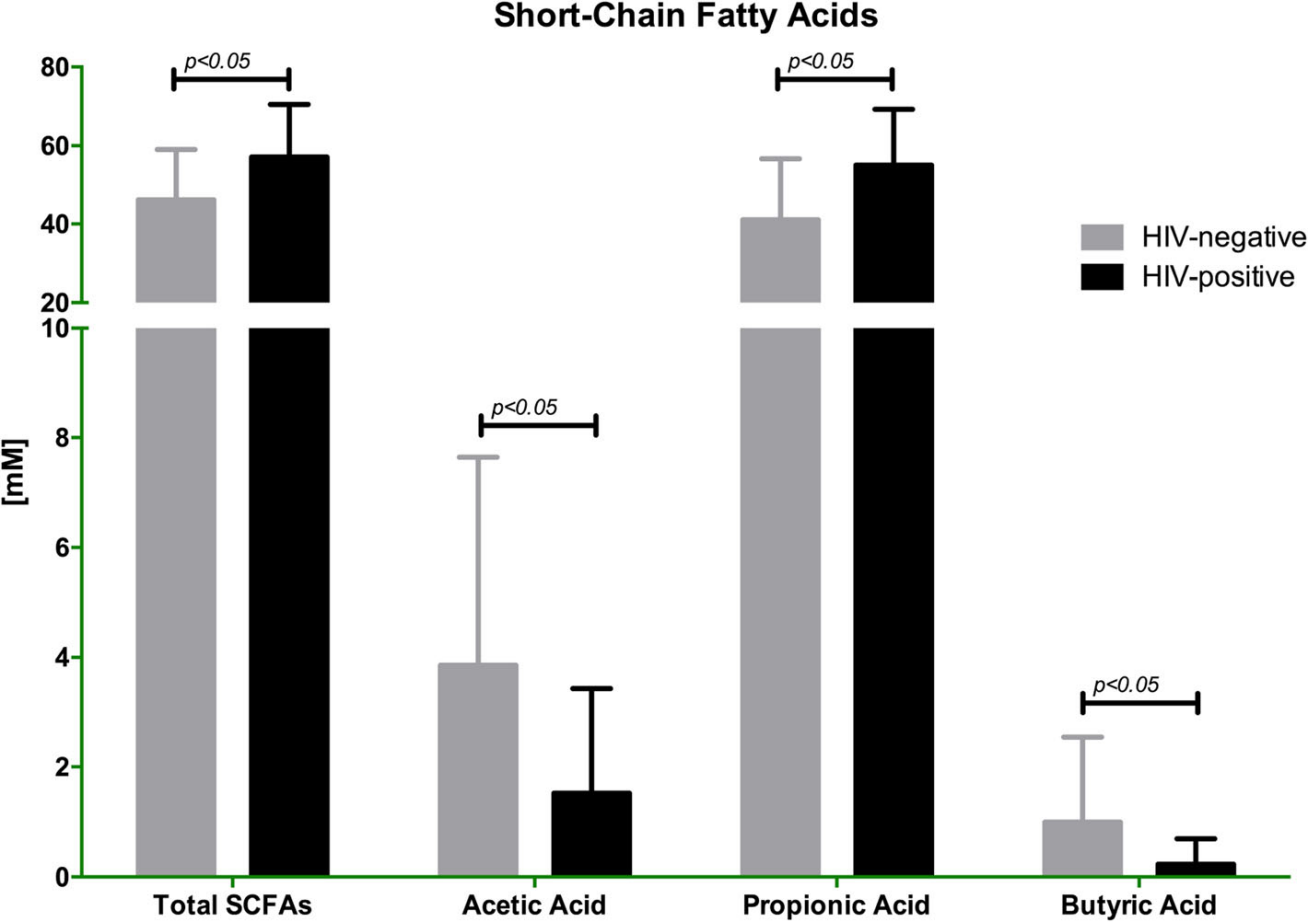
Fig. 3
